# Evaluation of *Metarhizium brunneum-* and *Metarhizium*-Derived VOCs as Dual-Active Biostimulants and Pest Repellents in a Wireworm-Infested Potato Field

**DOI:** 10.3390/jof9060599

**Published:** 2023-05-23

**Authors:** Martyn J. Wood, Alexandra M. Kortsinoglou, James C. Bull, Daniel C. Eastwood, Vassili N. Kouvelis, Pierre A. Bourdon, E. Joel Loveridge, Stephen Mathias, Abigail Meyrick, Audun Midthassel, Arben Myrta, Tariq Butt

**Affiliations:** 1 Department of Biosciences, Swansea University, Singleton Park, Swansea SA2 8PP, UK; 2 Institute of Molecular Biology and Biotechnology, Foundation for Research and Technology Hellas, 73100 Heraklion, Greece; 3 Department of Biology, Section of Genetics and Biotechnology, National and Kapodistrian University of Athens, 15772 Athens, Greece; 4 Department of Chemistry, Swansea University, Singleton Park, Swansea SA2 8PP, UK; 5 Puffin Produce Ltd., Withybush, Haverfordwest SA62 4BS, UK; 6 Certis Belchim BV, R & D Department, 3521 AZ Utrecht, The Netherlands

**Keywords:** *Metarhizium brunneum*, volatile organic compounds, potato, wireworm, repellent

## Abstract

Wireworm, the larval stages of click beetles, are a serious pest of tubers, brassicas and other important commercial crops throughout the northern hemisphere. No effective control agent has been developed specifically for them, and many of the pesticides marketed as having secondary application against them have been withdrawn from EU and Asian markets. *Metarhizium brunneum*, an effective entomopathogenic fungus, and its derived volatile metabolites are known to be effective plant biostimulants and plant protectants, although field efficacy has yet to be validated. Field validation of a combined *M. brunneum* and derived VOC treatments was conducted in Wales, UK, to assess the effects of each as a wireworm control agent and biostimulant. Plots were treated with Tri-Soil (*Trichoderma atroviridae*), *M. brunneum*, 1-octen-3-ol or 3-octanone, or combinations thereof. Treatments were applied subsurface during potato seeding (*n* = 52), and potatoes were harvested at the end of the growing season. Each potato was weighed individually and scored for levels of wireworm damage. Applications of both the VOCs and the *M. brunneum* individually were found to significantly decrease wireworm burden (*p* < 0.001). Combinations of *M. brunneum* and 3-octanone were also found to significantly decrease wireworm damage (*p* < 0.001), while no effect on yield was reported, resulting in an increased saleable mass over controls (*p* < 0.001). Herein, we present a novel ‘stimulate and deter’ wireworm control strategy that can be used to significantly enhance saleable potato yields and control wireworm populations, even under high pest pressure densities.

## 1. Introduction

Wireworms are the cryptic larvae of members of the click beetle family (Coleoptera: Elateridae). Within the UK, there are three species that are soil-dwelling agricultural pests known to cause significant damage: *Agriotes lineatus*, *Agriotes obscurus*, and *Agriotes sputator*, each of which have overlapping ranges and habitats. They are found in disparate habitats throughout the Northern Hemisphere [1], exhibiting intermittent feeding with extended non-feeding periods within the rhizosphere of host plants. They also exhibit vertical movement in the soil, burrowing deeper to avoid high summer and low winter temperatures [2]. Due to their intermittent feeding lifestyle and position deep within the soil, they are extremely difficult to control effectively [1]. They feed on a wide range of arable crops [3]. Potatoes are highly susceptible, and feeding damage can significantly reduce tuber quality and marketability [4,5].

To date, most control strategies for wireworms have focused on the use of insecticides [6], production of resistant plant varieties [7], and the integration of semiochemical compounds in ‘attract and kill’ strategies [8,9]. Due to environmental persistence and high toxicity, many of the traditionally used commercial insecticides, such as phorate and ethoprop, have been withdrawn from use in EU and Asian markets, including China and India, in the last decade [10], and they are expected to be withdrawn from US markets in the near future. Therefore, the development of benign control agents for use in cohesive IPM programmes is crucial for future sustainable crop production.

Entomopathogenic fungi (EPF) of the genus *Metarhizium* (Ascomycota: Hypocreales) are increasingly being used as environmentally friendly alternatives to traditional chemical pesticides [11]. Several strains have shown promise for the control of wireworms [8,12]. Furthermore, the fungus has demonstrated effective plant growth stimulation in a range of commercially valuable crops [13,14,15,16]. Altogether, these observations suggest a potential for *Mertarhizium* spp. to be used for the concomitant control of pests and promotion of plant growth, offering the potential to reduce inputs of conventional pesticides and fertilizers. 

While laboratory assessments of *M. brunneum* as a wireworm biopesticide have been encouraging, field translation is rarely achieved, with key problems associated with the depth-penetration requirements for the biocontrol agent to reach wireworms [17], strain dependent virulence against the target pest [18], and the soil structure inclusive of pH, inorganic matter content, and porosity, each of which affect the ability of the fungus to reach and infect the wireworm [8,19]. Effective screening of entomopathogens for virulence, formulation efficacy, and abiotic tolerance can, to some extent, help to mitigate these issues [20]. Further enhancements may also be possible through genetic engineering to enhance virulence factors [21] or use of selective media to increase fungal tolerance to abiotic stress, especially under field conditions [22].

Entomopathogenic fungi produce a diverse range of secondary metabolites, including antimicrobial compounds and volatile organic compounds (VOCs) with pesticidal and semiochemical properties [23,24,25,26,27]. Two key VOCs, 1-octen-3-ol and 3-octanone, produced by *M. brunneum* [27], have also been found to be highly effective plant biostimulants [15] alongside possessing insecticidal [28], nematicidal [29], and molluscicidal [30,31] properties. Furthermore, dose-dependent attractant/repellent properties of 1-octen-3-ol and 3-octanone have also been observed in other arthropod pests and vectors, including, but not limited to, several mosquitoes, grain beetles, collembola, or tsetse fly species [32,33].

Current biological control methodologies for managing wireworms involve the root-based application of bioinsecticides, with variable success rates [8,18]. Another strategy entails preseason cover crop inoculation with the intent of naturally establishing EPF in the rhizosphere to infect wireworms [34]. Unfortunately, the control levels did not sufficiently reduce potato damage to suggest that it was a sustainable control method. Furthermore, chemical insecticides applied simultaneously were also not found to reduce end-point damage to a significant degree. The failures of such strategies put the emphasis on more integrated methodologies, whereby the pest is controlled via simultaneous entomopathogenic fungal-induced mortality, alongside a strong semiochemical action that can prevent damage while the entomopathogens establish control.

Attract-and-kill approaches, using biopesticides in conjunction with semiochemical attractants, have also been tested for use against wireworms using CO_2_ capsules [8] and millet grain [35]. The control was successful in terms of lower crop damage, albeit damage was not completely mitigated for. Efficacy for these approaches may have been limited by the behaviour of the wireworm, as soil-swelling arthropods with low motility are less likely to respond to attractant baits over distance [36]. This is especially the case when longer distances exist between the target and the bait. Short range repellency, however, may cause a localised reduction in pest pressure around the plant, reducing feeding damage and overcoming the limitations of methods aimed at achieving attraction over distance. Given that *M. brunneum*-derived VOCs have shown potential as both wireworm repellents and as plant stimulants, the studies presented herein were devised to test field-scale proof of concept for a new form of IPM strategy, “stimulate and repel”, the concept being that low-dosage *M. brunneum* and formulated VOCs can be used in conjunction to simultaneously promote plant growth, repel wireworms, and offer broad biological control of the target pest, resulting in a significant reduction in damaged potato yield at end-point harvest. 

## 2. Materials and Methods

### 2.1. Maintenance of Fungal Cultures

*Metarhizium brunneum* strain V275 (commercial names: Met52, Bipesco 5, Lalguard) was cultured and maintained on Sabouraud Dextrose Agar (SDA) with bimonthly ‘passage’ through waxworm (*Galleria melonella*) larvae to ensure high virulence (i.e., to pre-empt attenuation). Conidia recovered from SDA cultures were suspended in 0.03% aqueous Tween 80 and 1 mL of 1 × 10^7^ conidia mL^−1^ used to inoculate 250 mL liquid Sabouraud Dextrose Broth (SDB) and incubated at 27 °C at 150 rpm for 3 days. Then, 50 mL aliquots of the mycelial broth were mixed into 500 g pre-soaked sterilised broken basmati rice and grown for 12 days at 27 °C. The rice was dried at room temperature for 3 days before sterile packaging in foil packets and the resultant conidia harvested using a mycoharvester. Harvested conidia were counted again using a haemocytometer and spores g^−1^ calculated prior to field assay. Conidia used in all assays were harvested < 2 weeks prior to the start of assay and stored at 4 ± 1 °C.

Commercially produced Tri-Soil (*Trichoderma atroviridae*) formulation was provided by Certis-Belchim BV (Utrecht, The Netherlands) 1 week prior to the start of the field trials.

### 2.2. VOC Production

Prototype VOC granules were prepared and provided by Certis-Belchim BV prior to the start of field trials.

VOC granules were prepared using a multilab granulator–extruder–spheronizer machine (Caleva, Sturminster Newton, England). Granules were prepared using 1-octen-3-ol and 3-octanone microcrystalline cellulose (MCC) (Table 1). MCC was the main carrier for each type of granule, as it helps with the formation of the granules [37]. The granules were prepared using the formulation developed by Japan Agro Services. All granules were loaded with 10% VOCs (wt:wt). The moisture content of the granules was ca. 28%. All granules were stored in foil-sealed plastic bottles for 2 weeks prior to the start of experimentation.

### 2.3. Potatoes

Seed potatoes (*Solanum tuberosum*; Var: Maris Piper) were provided by Puffin Produce Ltd. on the day of the trials. Potatoes had been stored over winter and were planted immediately on delivery. Thereafter, all growth took place under normal growing conditions in a newly ploughed commercial seed potato production field near Bosherston, Pembrokeshire, Wales, UK (GPS coordinates: 52.6228° N, 4.945° W), in a plot area measuring 240 m^2^. This site is known to have a very high wireworm burden.

### 2.4. Assessment of Efficacy of M. brunneum- and Metarhizium-Derived VOCs on Potato Yield in Wireworm (Elatiridae spp.)-Infested Field

Two key *Metarhizium*-derived VOCs identified as strong biostimulant compounds [15] were selected for enhanced assay to determine their end-point effects on potato yield alongside *Metarhizium brunneum* (strain: V275) and Tri-Soil (*Trichoderma atroviridae*, Certis-Belchim BV, Belgium) as known biostimulants (Table 1). Simultaneously, trials were designed to assess the impact of the VOCs on wireworm attack incidence in a field site with high wireworm burden.

**Figure 1:**
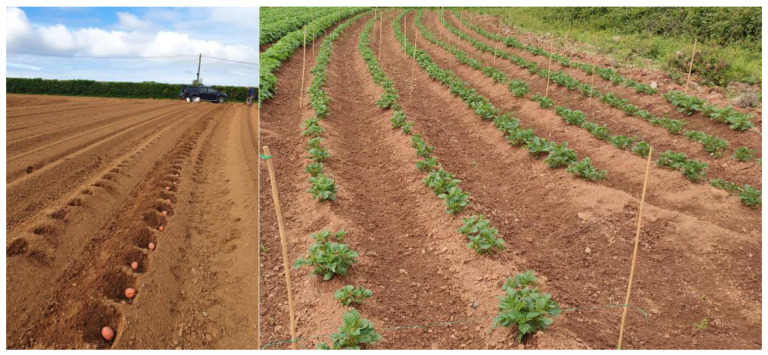
Images of trial site taken after 0 and 28 days. Left-hand image shows potatoes being planted in furrows above *M. brunneum* and derived-VOC treatments in subplots. Right-hand panel shows potato plant (Solanum tuberosum) growth four weeks post-planting. Trials took place at field periphery in Pembrokeshire, Wales, UK.

Unplanted furrows at the field periphery were used for manual application of treatments and seeding. Treatment blocks for each of the seven treatments (Table 1) were created measuring 1 m × 4 m, each consisting of two 4 m blocks within a furrow (Figure 1). At 30 cm intervals within each furrow, divots 25 cm deep were created for planting, giving a total of 13 potato plants per furrow and 26 plants per treatment block. At the base of each divot, treatments were applied at the specified rates (Table 1) before being covered with 5 mm of soil from the furrow edge. One seed potato was planted on top of the treatment and the furrow closed over thereafter. Normal farming practice, less the application of pesticides for wireworms, was continued throughout the trial period, inclusive of watering during drought periods experienced in June and July 2022. Trials were commenced in May 2022 and ran through to early September 2022, with the potato plants ‘topped’ in August to harden off the potatoes prior to harvest. Two experimental blocks were created for each treatment, giving a total of 52 potato plants for yield and damage analysis.

At the point of harvest, all potatoes were collected from the treatment blocks and pooled according to treatment. Potatoes were stored in paper potato bags in a cool and dark location (5 ± 2 °C) for up to 2 weeks post-harvest. Each potato was weighed individually and scored for wireworm damage. Scoring was conducted according to the protocols set by Puffin Produce Ltd. on a scale of 0–2 (0 = no wireworm damage, 1 = 1 wireworm attack/hole, 2 = multiple wireworm attacks/holes). Data derived from trials were designed to include: (i) total number of potatoes per 52 plants, (ii) mean number of potatoes per plant, (iii) mean mass of potatoes per plant, (iv) mean wireworm damage per potato, (v) size of potatoes vs. wireworm incidence, (vi) useable potato quantity and mass per treatment.

### 2.5. Statistical Analysis

The number of tubers was modelled as a zero-truncated generalised Poisson process. Mean weight per tuber and total weight summed over tubers (yield) were modelled as gamma processes. Damage was assessed as two binary responses: no damage vs. damage, score (0) vs. (1,2), and no damage or light damage vs. severe damage, (0,1) vs. (2), modelled as binomial processes. Each of these scenarios was assessed as a separate Generalised Linear Mixed Model (GLMM). The effects of ‘Treatment’ and potato ‘Weight’ on the probability of potato damage were fitted as interacting fixed effects. Fixed effects were nested within experimental ‘Block’ through the random effects structure of the model. Overall effects of predictor variables were assessed using likelihood ratio (L.R.) tests, and multiple pairwise comparisons between treatments were adjusted for familywise error rate using the Tukey post hoc method. Statistical modelling was performed using R version 4.2.2 (R Core Team, 2022. Vienna, Austria). GLMMs were developed using the glmmTMB package, with marginal means extracted using the emmeans package and graphs produced using the ggplot2 package.

## 3. Results

### 3.1. Effects of Metarhizium brunneum and Its Derived VOCs on Potato Yield Metrics

**Figure 2:**
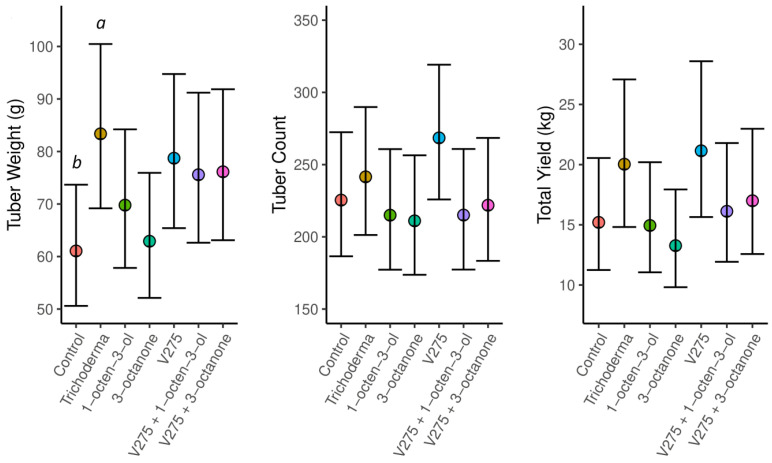
Left-hand panel. Mean weight (g) per potato tuber. Middle panel. Total number of tubers per block (26 plants) during the course of the field trial. Right-hand panel. Total potato yield (kg) per block (26 plants). Values are marginal means and 95% confidence intervals from GLMMs. Letters above given value scores denote significance between treatments (*p* < 0.05) whereby letters (a–b) represent untreated control (a) and *Trichoderma* treatment (b).

The association between VOC treatment and mean potato tuber weight was statistically significant (Figure 2, L.R. = 63.9, *p* < 0.001), but the association between treatment and total potato tuber count at harvest at the end of the growing season was not statistically significant (L.R. = 0.009, *p* = 0.996). When looked at in combination (Figure 1), for some treatments, an increase in tuber weight compared to controls (Table S1) was offset by a decrease in tuber number (1-octen-3-ol and 3-octanone), whereas for other treatments, an increase in both individual weight and number was recorded (*Trichoderma* and V275). However, due to relatively substantial variation between replicates, the overall association between VOC treatment and potato yield was not statistically significant (L.R. = 5.72, *p* = 0.456).

Potato tuber weight was enhanced most (37% over control samples) with *Trichoderma* treatment (Gamma GLMM: z = 2.31, *p* = 0.021). Importantly, no VOC treatments significantly reduced potato output, compared to controls (Table S1). Mean tuber weight was significantly increased only in plants treated with Tri-Soil (*T. atroviridae* T1237); in other experiments, no significant differences were found between treatments and controls (Table S2). Harvested tuber quantities were found to follow similar trends. No significant differences were found between treatments, although in terms of total numbers, Tri-Soil treatments produced the greatest metrics, closely followed by *M. brunneum* V275 as a sole treatment (Table S3).

### 3.2. Effects of Metarhizium brunneum and Its Derived VOCs on Wireworm-Associated Damage to Potato Tubers

**Figure 3:**
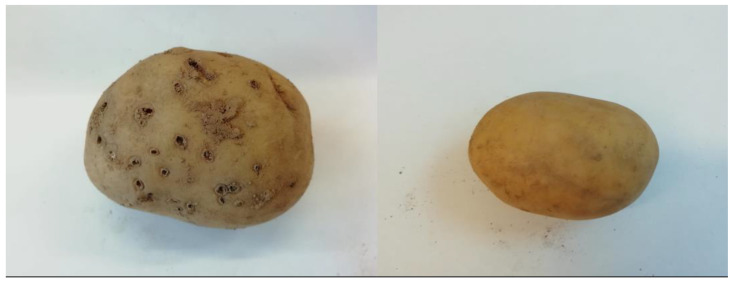
Images showing severity of wireworm damage in harvested potatoes. Left-hand panel shows potato tuber taken from control plot at harvest with multiple wireworm attack holes present on surface of potato. Right-hand panel shows unaffected potato retrieved from *M. brunneum* and 3-octanone combination treatment plot.

**Figure 4:**
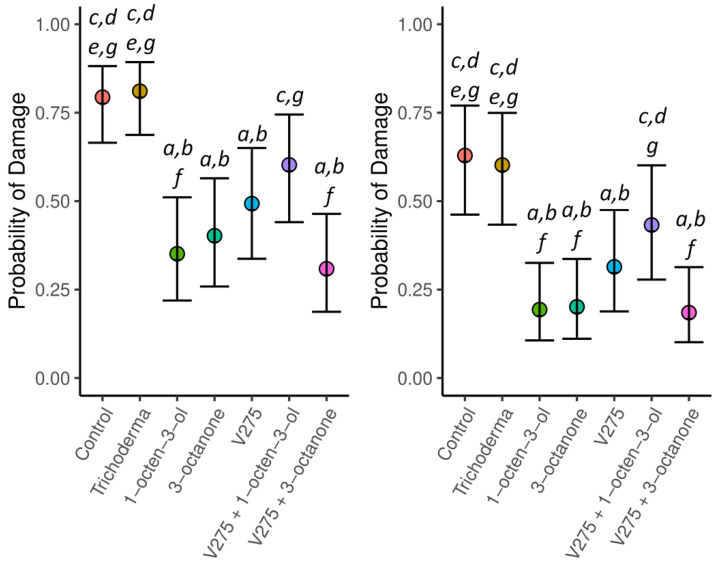
Mean relative wireworm damage scores for potatoes treated with VOCs, *Trichoderma*, and *M. brunneum* conidia, or combinations thereof, during early plant growth phases. Scoring was relative to wireworm damage incidence in harvested potatoes (0 = no wireworm damage, 1 = 1 wireworm attack/hole, 2 = multiple wireworm attacks/holes). Left-hand panel. No damage [score 0] vs. damage (1,2). Right-hand panel. No damage or light damage (0,1) vs. severe damage (2). Values are marginal means and 95% confidence intervals from GLMMs. Letters above given values denote significance between treatments (*p* < 0.05), whereby letters (a–g) represent each treatment reading sequentially from left to right.

Wireworm damage was found to be extensive throughout the trial site (Figure 3). Scored assessments of wireworm damage per treatment also produced statistically significant results (score (0) vs. (1,2): L.R. = 444, *p* < 0.001. Score (0,1) vs. (2): L.R. = 399, *p* < 0.001). Both VOCs, 1-octen-3-ol (*p* < 0.001) and 3-octanone (*p* < 0.001), were found to significantly reduce mean damage scores, as opposed to control samples using GLMMs (Figure 4). *Metarhizium brunneum* conidia applied as a sole agent also significantly reduced wireworm damage as compared to controls (*p* = 0.001), and wireworm damage following *Metarhizium brunneum* treatment was not significantly greater than for treatments using either VOC as a sole agent (*p* > 0.05 in both cases). A combination of *Metarhizium* with 3-octanone was more effective in significantly reducing mean wireworm damage when compared with the untreated controls and Tri-Soil (*p* < 0.001 in both cases) and approximately equal to 3-octanone as an individual treatment (*p* = 0.902). By contrast, combination treatments of 1-octen-3-ol and *M. brunneum* conidia acted antagonistically, with an increase in wireworm damage over 1-octen-3-ol (*p* = 0.044) and no improvement compared to *M. brunneum* (*p* = 0.848) as individual treatments. The damage recorded for these combinations was, however, still lower than that recorded for controls (*p* < 0.001), with the exception of *M. brunneum* and 1-octen-3-ol combined (*p* = 0.100).

Finally, we assessed the effect of potato weight on wireworm burden, as well as whether different treatments affected this relationship (statistical interaction). We found a positive relationship between wireworm burden and potato weight increase (1.3% (SE = 0.08%) in the probability of damage per additional gram of tuber weight (z = 14.39, *p* < 0.001). However, this relationship was independent of treatment (statistical interaction L.R. = 12.87, *p* = 0.075).

## 4. Discussion

This study demonstrates, for the first time, the potential of a novel ‘stimulate and repel’ strategy that could be integrated into wireworm management strategies. It was found that treatment of potato furrows during planting with combinations of *M. brunneum* and VOCs caused a significant reduction in wireworm damage to tubers at harvest. In particular, combinations of *M. brunneum* and 3-octanone were efficacious and were not found to produce any adverse effects on the yield obtained, even under the high wireworm pressure present in the study site. 

The wireworm burden on key crops has necessitated the development of novel strategies, particularly after the withdrawal from the market of the majority of its relevant control agents [6,10], inclusive of the dual-action pesticide MOCAP used initially for plant parasite nematode control. Somewhat effective ‘lure and kill’ strategies have been developed for wireworms [8,9]; however, given the limited mobility of wireworms, these strategies’ potential is limited in high-burden fields. Additionally, they do not offer additional protections against other forms of pest and disease burden. 

Both *M. brunneum* and the VOCs have been successfully demonstrated in laboratory studies to be multi-action plant protection and growth enhancement products [15]. *Metarhizium anisopliae*, a related EPF, has shown variable efficacy in controlling wireworms. Kabaluk and Ericsson (2007) showed that *M. anisopliae* (Strain: F52)-treated seeds resulted in improved development and an enhanced crop yield in maize [38]. Furthermore, it resulted in a significant increase in stand density potential. While Kabaluk and Ericsson (2007) found good control of wireworms during the trial, accounts of the efficacy of the F52 strain are variable, with Reddy et al. 2014 finding seed treatment with the same strain to fail to achieve satisfactory control levels, especially in areas where wireworm density was considerable [18,38]. This disparity may be due to the different wireworm species that formed the focus of each study, suggesting that individual wireworm species possess different levels of susceptibility, while other environmental factors including soil type can have a significant effect on the control levels achieved [19].

In the present study, application of *M. brunneum* at the potato seed stage is demonstrated to be an adept plant protection product, offering a significant level of pest damage mitigation against wireworms, lending support to the conclusions drawn in Kabaluk and Ericsson (2007) [38] and Ensafi et al. (2018) [19]. The VOCs had extremely high efficacy as wireworm repellents and damage deterrents in the field. The combination of the two appears to take the best characteristics of each product, resulting in a significant degree of increased potential saleable mass, even in fields with high pest burdens. Furthermore, these VOC products have been found to confer several other advantages, including plant growth stimulation [15], alongside nematicidal [29], insecticidal, and insect-repellent properties [28]. Given the broad scope of potential action for these VOCs, it may be that the ‘stimulate and deter’ strategy described herein can be expanded for use against a range of pest organisms other than wireworms, forming part of a cohesive multi-action IPM strategy in situations where a more complex pest-pressure burden is present. *Metarhizium brunneum* and its VOCs are effective plant growth stimulants [15], with demonstrated efficacy as control agents and semiochemicals for a range of soil-dwelling arthropods [18,28,39]. The mechanisms of these actions remain poorly understood, and data-driven studies, to date, are only able to infer the potential regulating mechanisms. *Trichoderma atroviridae* has been previously found to increase yield as a potato plant endophyte in glasshouse assays without insect pest burden [40]. Similarly, *M. brunneum* has been found to enhance potato plant vitality and growth as an endophyte [41]. In our study, significance was only found for an increase in mean tuber weight for those plots treated with *T. atroviridae*, despite outright mean values for tuber production and weight being higher than controls for plants treated with either fungus. Given that both treatments produced similar outright means, and that *T. atroviridae* is not an entomopathogen, it is likely that the benefits incurred to the plants go beyond simple pest control that could be attributable to *M. brunneum* and a multitude of rhizosphere effects. These may include endophytic benefits, even perhaps the production of metabolites and VOCs with repellent properties in and of themselves; *T. atroviridae* VOC bouquets are known to change as they form an endophytic association [40], and *M. brunneum* has even been found to produce specific pest-repellent VOCs [18,42]. Given the lack of recovered cadavers, it is possible that the development of repulsive compounds as plant secondary metabolites contributed to the reduction in damage seen in plants treated with *Metarhizium* only, meaning that direct entomopathogenic activities may not have been solely attributable to damage mitigation. Indeed, while combinations of *M. brunneum* with 3-octanone produced significant levels of control when compared to untreated controls, combinations of *M. brunneum* with 1-octen-3ol were not found to have the same effect, despite 1-octen-3-ol being an effective damage mitigant as a sole treatment. The suggestion would be that there is some antagonism between the *M. brunneum* and the 1-octen-3-ol in the present study. 1-octen-3-ol is a known inhibitor of fungal growth [43], and it is possible that the high presence of the compound within the soil environment mitigated *Metarhizium* growth so as to limit wireworm damage mitigation, potentially through decreased metabolic alteration to the plants when compared to those treated with an effective *M. brunneum* inoculation.

This is the first study to demonstrate that the entomopathogenic fungus *M. brunneum* can be combined with additive treatments of derived VOCs—1-octen-3-ol and 3-octanone—to successfully control wireworms in the field. Furthermore, this is the first study to demonstrate that these plant protection products, previously defined in laboratory and glasshouse studies, can translate into ‘real world’ enhancements to agricultural product saleable mass. We conclude that the use of a ‘stimulate and deter’ strategy forms a highly adept new tool for the integrated management of wireworm burden in commercial agricultural production. With further development, formulation, and elucidation of the mechanisms at play, this tool could be of high significance in the development of all-encompassing wireworm control strategies for future agri-tech. 

## 5. Conclusions

We conclude that *Metarhizium brunneum* and the *M. brunneum*-derived VOCs, 1-octen-3-ol and 3-octanone, show significant promise as wireworm control agents in a novel ‘stimulate and deter’ strategy. The data presented demonstrate the consistency of prior works and add to the knowledge base by providing a field evaluation under the context of heavy wireworm pest pressure. Applications under these circumstances led to what would be a significant increase in saleable yield, thereby constituting a new component that can be used within integrated pest management strategies in farms with a pest complex containing wireworms, traditionally a very difficult pest to control.

## Figures and Tables

**Table 1:** Treatment descriptions for assays using *M. brunneum* strain V275—all treatments applied as subsurface granular applications.

Treatments	Active Ingredient, Manufacturer	Dose Rate
T1: Untreated Control	N/A	NA
T2: Tri-Soil	*Trichoderma atroviridae*	5 kg/Ha
T3: 1-octen-3-ol granules	1-octen-3-ol (10% *w*/*w*)	30 kg/Ha
T4: 3-octanone granules	3-octanone (10% *w*/*w*)	30 kg/Ha
T5: *M. brunneum* (V275)	*M. brunneum* conidia	1 × 10^9^ conidia/plant
T6: V275 + 1-octen-3-ol granules	*M. brunneum* + 1-octen-3-ol (10% *w*/*w*)	1 × 10^9^ conidia/plant + 30 kg/Ha
T7: V275 + 3-octanone granules	*M. brunneum* + 3-octanone (10% *w*/*w*)	1 × 10^9^ conidia/plant + 30 kg/Ha

## Data Availability

Data is openly available via Figshare. DOI: 10.6084/m9.figshare.23065523.

## Supplementary Materials

The following supporting information can be downloaded at: https://www.mdpi.com/article/10.3390/jof9060599/s1, Table S1: Mean potato mass (g) for each potato recovered from included treatments. Total harvested potatoes weighed and averaged according to total tuber number; Table S2: Total potato count tuber for each treatment. Total number of harvested potatoes were weighed individually in pooled per block (divide treatment means by 26 for per plant means); Table S3: Total potato yield (mass; g) for each treatment. Total number of harvested potatoes were weighed individually in pooled per block (divide treatment means by 26 for per plant means).
