# Supplementary material for: Evaluation of Metarhizium brunneum- and Metarhizium-Derived VOCs as Dual-Active Biostimulants and Pest Repellents in a Wireworm-Infested Potato Field
Source: J Fungi (Basel). 2023 May 23;9(6):599. doi: 10.3390/jof9060599 (PMC10301102; doi:10.3390/jof9060599)
Supplement: Supplementary file 1 [file jof-09-00599-s001.zip › jof-2411159-supplementary.pdf]

Table S1. Mean potato mass (g) for each potato recovered from included treatments. Total harvested potatoes weighed and averaged according to total tuber number.

	Mean	SE	Lwr 95% ci	Upr 95% ci
Control	61.06	5.852	50.6	73.683
Trichoderma	83.368	7.934	69.177	100.47
1-octen-3-ol	69.785	6.691	57.825	84.218
3-octanone	62.912	6.039	52.119	75.941
V275	78.722	7.442	65.402	94.754
V275 + 1-octen-3-ol	75.575	7.246	62.624	91.205
V275 + 3-octanone	76.135	7.29	63.103	91.857

Table S2. Total potato count tuber for each treatment. Total number of harvested potatoes were weighed individually in pooled per block (divide treatment means by 26 for per plant means).

	Mean	SE	Lwr 95% ci	Upr 95% ci
Control	225.428	21.783	177.959	285.559
Trichoderma	241.535	22.471	192.36	303.28
1-octen-3-ol	214.988	21.167	168.961	273.552
3-octanone	211.078	20.963	165.541	269.141
V275	268.523	23.681	216.404	333.195
V275 + 1-octen-3-ol	215.064	21.169	169.031	273.634
V275 + 3-octanone	221.884	21.58	174.893	281.5

Table S3. Total potato yield (mass; g) for each treatment. Total number of harvested potatoes were weighed individually in pooled per block (divide treatment means by 26 for per plant means)..

	Mean	SE	Lwr 95% ci	Upr 95% ci
Control	15201.168	2338.151	10433.306	22147.87
Trichoderma	20031.427	3081.111	13748.551	29185.48
1-octen-3-ol	14942.42	2298.353	10225.713	21770.884
3-octanone	13268.442	2040.871	9106.78	19331.92
V275	21148.535	3252.938	14515.276	30813.092
V275 + 1-octen-3-ol	16120.521	2479.561	11064.302	23487.354
V275 + 3-octanone	16997.661	2614.476	11666.329	24765.331
